# Modified biplanar (0-90^o^) endoscopic-guided puncture technique for percutaneous nephrolithtomy: refinement with endoscopic combined intrarrenal surgery to reduce fluoroscopy and operative time

**DOI:** 10.1590/S1677-5538.IBJU.2023.0346

**Published:** 2024-02-07

**Authors:** Giovanni Scala Marchini, Fábio Sepúlveda Lima, Marcelo Esteves Chaves Campos, Marcus Vinícius Osorio Maroccolo, Ernesto Reggio, Eduardo Mazzucchi, William Carlos Nahas, Luiz Sérgio Santos, Thiago Hota

**Affiliations:** 1 Faculdade de Medicina da Universidade de São Paulo Hospital das Clínicas Divisão de Urologia São Paulo SP Brasil Seção de Endourologia, Divisão de Urologia, Hospital das Clínicas, Faculdade de Medicina da Universidade de São Paulo, São Paulo, SP, Brasil;; 2 Hospital do Idoso Zilda Arns Curitiba PR Brasil Endouroexperts - Hospital do Idoso Zilda Arns, Curitiba, PR, Brasil

## Abstract

**Introduction:**

We aim to publish our innovative modified biplanar 0-90 endoscopic guided puncture technique for percutaneous nephrolithotomy in supine recorded with a GoPro^®^ camera for standardization of the technique. It solves drawbacks of the fluoroscopic technique, i.e., in kidneys with complex anatomy, it may be challenging to distinguish calyces as they are often superposed, and it does not allow for all benefits of a combined endoscopic approach (1, 2). Our technique shortens puncture and fluoroscopic time and is easy to teach and reproduce.

**Methods:**

A 77-year-old female patient had previous double J insertion due to an obstructing stone in the right distal ureter. She managed to pass the distal stone but remained with the double J and a 20mm stone (1300HU) in the right renal pelvis. The shared decision was for the actual standard of care (3, 4) endoscopic combined intrarenal surgery (ECIRS). The MiniECIRS started with flexible ureteroscopy and a posterior calix which gave direct access to the stone was chosen. The tip of the flexible scope was used to mark point A with the C-arm in the 0-degree position and line B in the 90-degree position. Puncture was fast and the MiniECIRS was uneventful with a single mid-pole access guided by the flexible scope. The surgeon had a Full-HD GoPro^®^ camera mounted on his head, controlled by the surgical staff. All essential surgical steps were recorded.

**Results:**

The quality of the recorded movie was graded as excellent, and the camera did not cause any discomfort to the surgeon. Operative and X-Ray time were 120minutes and 2minutes (7.64mGy). Hemoglobin drop was 0.8g/dL. The post-operative day-1 computed tomography scan was stone-free. The patient was discharged 24h after surgery. Kidney stent was left with a string and removed after 5days. The patient remained asymptomatic and metabolic evaluation revealed a calcium oxalate stone, low urinary volume and hypocitraturia which were treated with potassium citrate and hydration.

**Conclusion:**

The Modified Biplanar (0-90 degree) Endoscopic-Guided Puncture Technique for Percutaneous Nephrolithotomy joins the reproducibility of the same technique under fluoroscopy with advantages regarding safety and efficiency of ECIRS.

**Available at:** http://www.intbrazjurol.com.br/video-section/20230346_Marchini_et_al

**Int Braz J Urol. 2023; 49 (Video #14): 785-6**
